# Hydrocephalus and Papilledema in Spinal Cord Tumors: A Report of Two Cases

**DOI:** 10.1055/s-0036-1584584

**Published:** 2016-06-29

**Authors:** Ahmad Nabil Marzban, Ankur Saxena, Dev Bhattacharyya, Marcel Ivanov

**Affiliations:** 1 Department of Orthopedics and Spine Surgery, Cairo University, Cairo, Egypt; 2 Department of Neurosurgery, Salford Royal Hospital NHS Foundation Trust, Manchester, United Kingdom; 3 Department of Neurosurgery, Royal Hallamshire Hospital, Sheffield Teaching Hospitals NHS Foundation Trust, Sheffield, United Kingdom

**Keywords:** spinal cord tumors, intraspinal, hydrocephalus, papilledema, increased intracranial tension

## Abstract

**Background** The association between spinal cord tumors and hydrocephalus is a rarely reported phenomenon. Diagnosis in this group of patients is difficult as they present with findings of an intracranial pathology and the symptoms of a spinal lesion may be absent.

**Case Report** We report two cases of spinal cord tumors presenting with visual disturbance and findings of increased intracranial pressure.

**Discussions** Mechanisms describing the relationship between spinal cord tumors and increased intracranial pressure have been explained. Most of the literature reported marked regression of these manifestations after tumor excision.

**Conclusions** Spinal cord tumors associated with hydrocephalus and papilledema are rare conditions. The diagnosis of these conditions may be difficult or confusing because the symptoms referable to the spinal lesion may be minimal. Meticulous history taking, examination, and investigations are mandatory to diagnose this entity.

The association between spinal cord tumors with hydrocephalus and papilledema is rare with ∼1% of patients with spinal cord tumors presenting with hydrocephalus. 1 2 Hydrocephalus associated with spinal cord tumors was mentioned in ∼300 reports in the literature. 3 4 The first one was described in 1931 by Kyrieleis, and the patient was cured after removal of the tumor. 5 Papilledema was reported in only about nine cases until 2009. 2 6 7 8 Spinal astrocytomas and ependymomas are more frequently associated with hydrocephalus, 3 with ependymomas forming more than 50% of these tumors. Also, papilledema-associated spinal cord tumors were noted to be located at lower spinal levels, usually in the thoracolumbar or lumbosacral regions. 9 Diagnosis in this group of patients was challenging because the patients presented with findings suggesting an intracranial pathology; the symptoms referable to the spinal lesion were subtle, and some of them were initially overlooked. 7 In this report of two cases, we stress the importance of proper assessment of patients presenting with manifestations of increased intracranial tension without an intracranial pathology, highlighting the entity of spinal cord tumors.

## Case Reports

### Case One

**Fig. 1:**
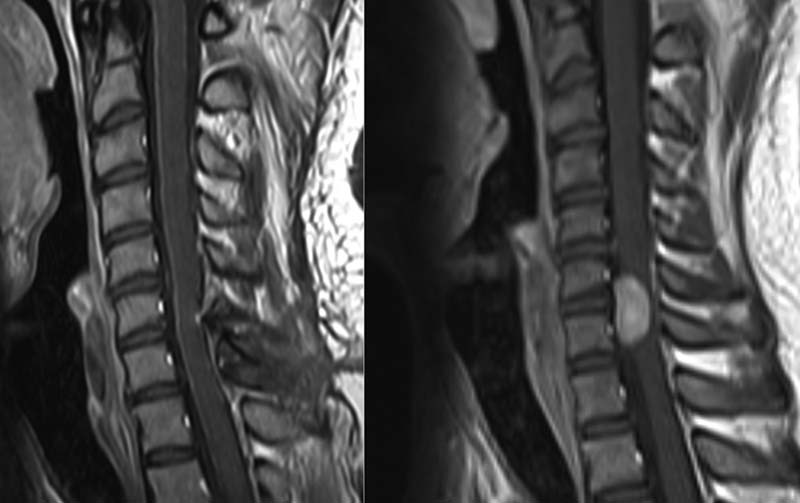
Sagittal T1-weighted magnetic resonance imaging of the first case preoperatively (right) showing a bright signal denoting an intradural extramedullary tumor and 1 month postoperatively (left) showing complete removal.

**Fig. 2:**
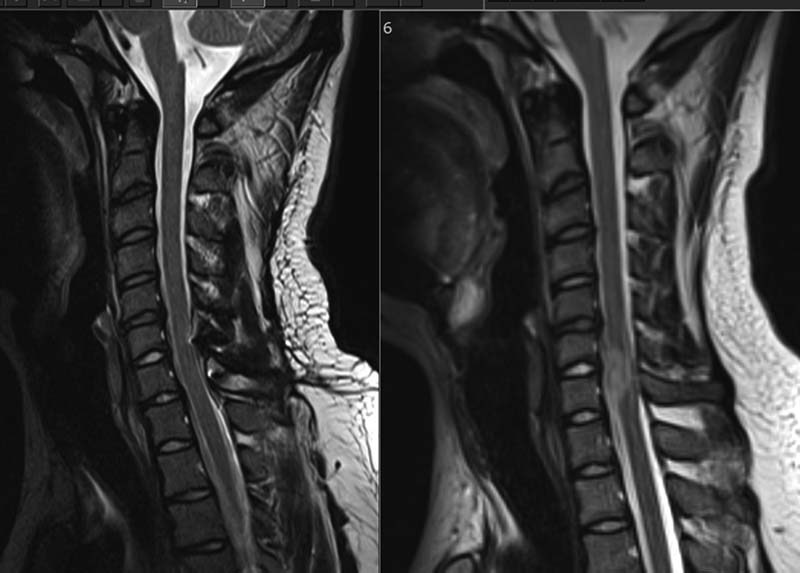
Sagittal T2-weighted magnetic resonance imaging of the first case showing intermediate signal of the lesion preoperatively (right) and postoperatively (left).

A 22-year-old man presented to the ophthalmology clinic complaining of headache and blurred vision for 4 weeks. He did not have any significant past medical history and was not on any regular medication. On examination, his visual acuity was 6/5 on the right and 6/12 on the left. Fundus examination showed bilateral papilledema worse on the left side. Bulk, tone, power, and sensation were normal in all limbs but his reflexes were brisk on the right side. Computed tomography and magnetic resonance imaging (MRI) of the brain did not reveal any abnormality. A lumbar puncture was performed to obtain a sample for analysis and culture to exclude infection. The opening pressure was 46.5 cmH _2_ O of cerebrospinal fluid (CSF). Investigations of the CSF revealed a glucose level of 3.2 mmol/ L and protein level of 1.53 g/L. CSF cultures ruled out infection. MRI of the patient's spine was requested in view of the increased CSF protein level, which could be attributed to a vertebral or an intradural pathology, and showed an intradural extramedullary tumor opposite the sixth and seventh cervical vertebrae ( Figs. 1 and 2 ) *.* Surgery was performed for excision of the tumor. Postoperatively, there was reduced sensation of the right hand, especially the fingertips, with mild weakness of the left leg and decreased mobility.

**Fig. 3:**
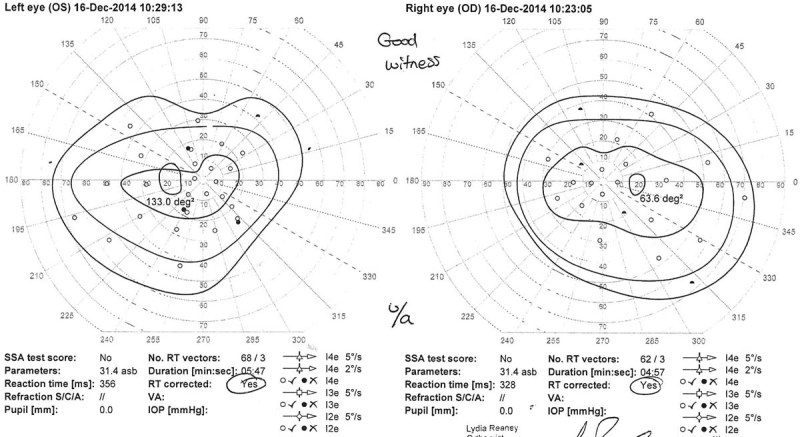
Visual field perimetry of the first case before surgery showing enlargement of the blind spot mainly on the left side.

**Fig. 4:**
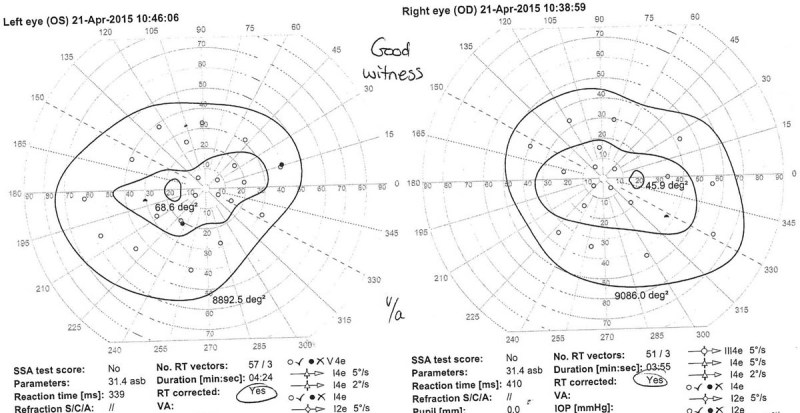
Visual field perimetry of the first case 4 months postoperatively showing improvement in the blind spot especially on the left side.

Histopathologic examination confirmed a World Health Organization grade I schwannoma. Postoperative MRI performed a month later showed no residual mass or recurrence. At 2 months following surgery, the patient was independently mobile and completely asymptomatic, with improvement in the visual fields ( Figs. 3 and 4 ). Three months after surgery, the visual acuity was 6/4 on the right and 6/6 on the left side. The plan is for regular clinical and radiologic surveillance on a yearly basis.

### Case Two

**Fig. 5:**
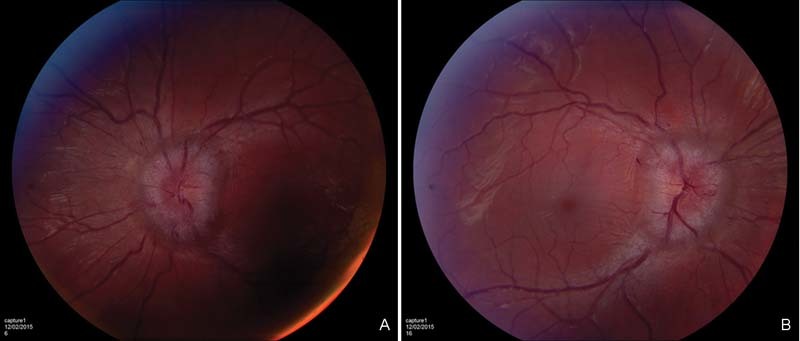
Photos of fundus examination of the second case showing bilateral papilledema with slight enlargement of the blind spot and nasal constriction in (A) right and (B) left eyes.

**Fig. 6:**
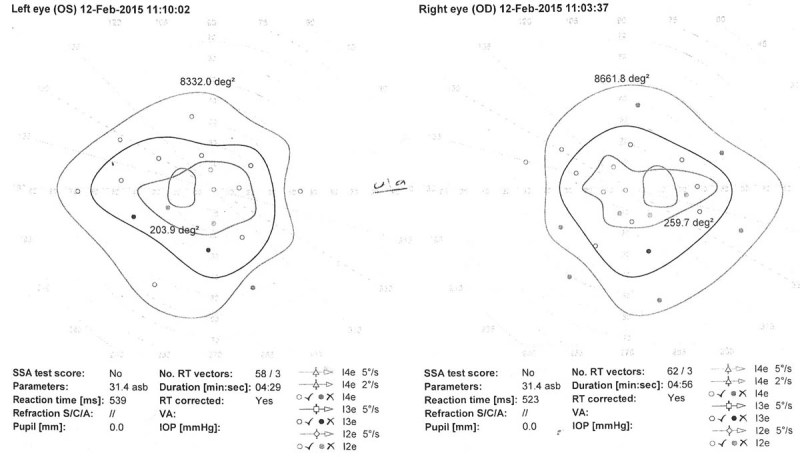
Preoperative visual field perimetry of second patient showing enlarged blind spot bilaterally.

A 19-year-old man with neurofibromatosis type I presented with blurring of vision and diplopia of 3 weeks' duration and progressively worsening midthoracic pain for 1 year. On further questioning, the patient reported difficulty in walking with both legs feeling weak, heavy, and numb. On examination, there was marked kyphosis of the cervical spine. The visual acuity was 6/9 on the right and 6/6 on the left side. Fundus examination showed bilateral papilledema with slight enlargement of the blind spot and nasal constriction ( Fig. 5 ). Visual field perimetry showed enlargement of the blind spot bilaterally ( Fig. 6 ).

**Fig. 7:**
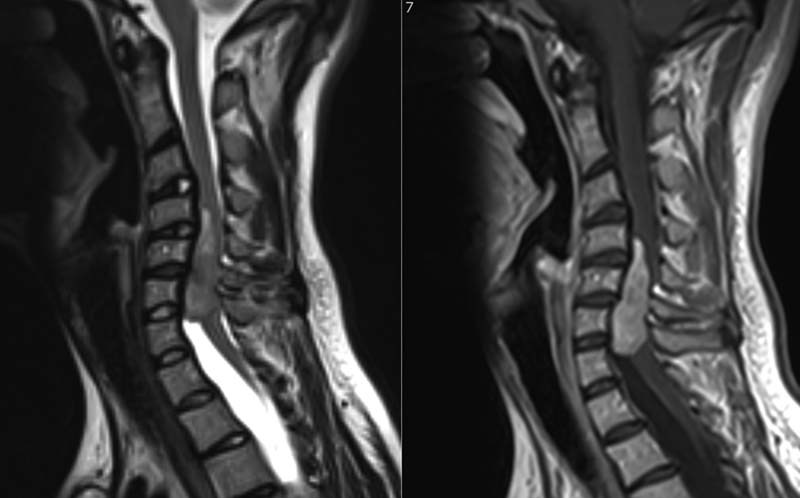
Sagittal (left) T2-weighted and (right) T1-weighted magnetic resonance imaging of the second case showing an intradural extramedullary tumor with intermediate and bright signal intensity, respectively, extending opposite fourth to seventh cervical vertebrae.

**Fig. 8:**
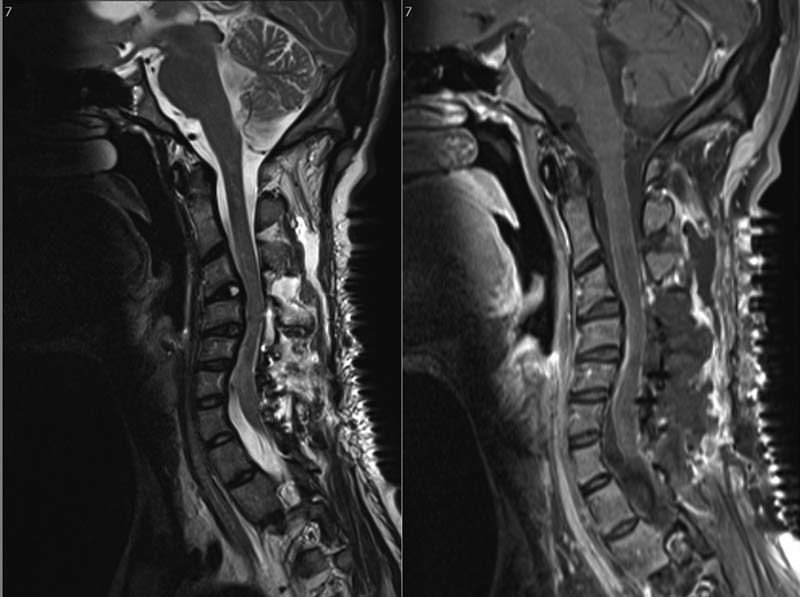
Sagittal (left) T2-weighted and (right) T1-weighted postoperative magnetic resonance imaging of the second case showing complete removal of the tumor.

**Fig. 9:**
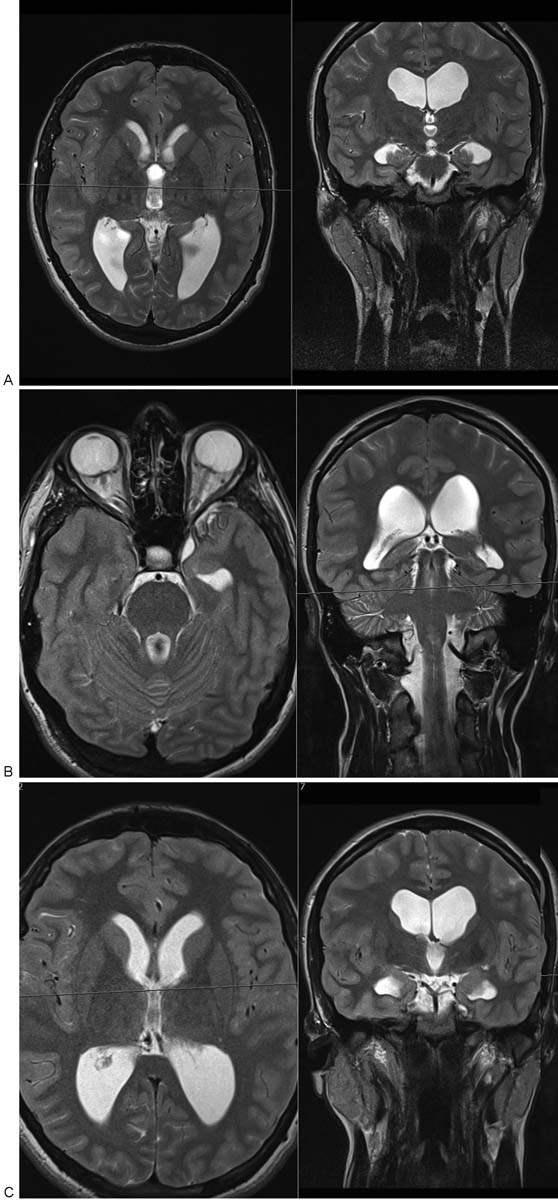
(A, B, C) T2-weighted magnetic resonance imaging of the brain in the second case demonstrates nonobstructive hydrocephalus with enlargement of all four ventricles.

No other cranial nerve deficit was noted. Peripheral neurologic examination revealed hypertonia with normal power in all four limbs, a sensory level at T4–T6, and hyperreflexia with clonus. An MRI of his brain and spine showed an intradural extramedullary tumor extending opposite the fourth to seventh cervical vertebrae causing spinal cord compression ( Figs. 7 and 8 ). Ventriculomegaly was noted, confirming the clinical suspicion of hydrocephalus ( Fig. 9 ).

The patient underwent surgery for excision of the tumor through C4 to C6 laminectomy without fusion, and there were no intraoperative complications. The postoperative recovery was complicated by continuous CSF leakage through the wound that did not subside with further sutures. The patient eventually required a ventriculoperitoneal shunt because hydrocephalus was thought to be the cause of CSF leakage. After shunt insertion, the wound remained dry. The patient required a significant amount of spinal rehabilitation for recovery because he suffered from residual weakness and sensory loss in both his legs after surgery. The histopathologic examination was positive for melanin antibody, which raised the possibility of the benign end of the spectrum of primary melanocytoma or a neurocutaneous syndrome. The patient was referred to a dermatologist for further care.

## Discussion

Spinal cord tumors can cause raised intracranial pressure with or without the radiologic presence of ventriculomegaly. 6 The pathophysiology of increased intracranial tension may differ according to the type of spinal cord tumor. Malignant tumors can increase CSF outflow resistance because of their natural tendency to spread in the subarachnoid pathways (meningeal infiltration). Several pathophysiological theories have been proposed with benign lesions. 3 Some authors believe the lesions attribute to increased CSF viscosity due to elevated CSF protein level, which in turn decreases CSF absorption at the arachnoid villi. However, a high protein level was not always found in cases of hydrocephalus due to intraspinal tumors. It was also shown that high protein concentrations did not greatly affect CSF viscosity. These findings therefore do not support this theory. 3

The most likely explanation may be release of a tumor-generated chemical into the CSF that hinders CSF absorption. Removal of the tumor will lead to normalization of intracranial pressure. Several potential chemical markers have been suggested. One is fibrinogen, which is converted to fibrin in the CSF. If a blockage occurred at the basal cisterns, hydrocephalus would result, but if the blockage occurred at the level of the arachnoid villi, it could lead to the pseudotumor cerebri syndrome. 10 Increased CSF fibrinogen could be the result of a chronic inflammatory response, breakdown of the blood–brain barrier, or a subarachnoid hemorrhage. It was supported by CSF coagulation in a sample tube (Froin syndrome), the occurrence of hydrocephalus after an acute or repeated subarachnoid hemorrhage in some spinal tumors, and finally the experimental use of recombinant tissue plasminogen activator to treat subacute hydrocephalus in experimental subarachnoid hemorrhage. 3 Another interesting chemical is the inflammatory cytokine transforming growth factor-β. Found in a variety of primarily vascular structures at the choroid plexus, cytokine transforming growth factor-β is present in high concentrations in platelets and has been shown to result in the proliferation of leptomeningeal cells and the creation of scarring both at the base of the brain and in the area of the arachnoid villi. 11

According to the hydrodynamic theory, Morandi et al described hydrocephalus in patients with benign intraspinal tumors possibly caused by a reduction in the spinal compliance secondary to tumoral obstruction of the spinal subarachnoid space that reduced total CSF compliance, which may lead to hydrocephalus due to a “water hammer effect.” 4 12 The reduction in spinal compliance is partially compensated for by the vascular pool, which if not sufficient may cause an increase in mean intracranial pressure. 7

Neoplastic arachnoiditis in cases of malignant intraspinal tumors and hydrocephalus could be explained on a pathophysiological basis as subarachnoid dissemination, and widespread meningeal tumoral infiltration has been documented in the majority of these cases. 7

The same theories apply in the pathogenesis of papilledema in spinal tumors: excessive protein secretion as well as mechanical obstruction of the CSF flow by the spinal tumor can increase CSF viscosity, which may affect the axoplasmic flow directly, causing disk edema, 8 or indirectly through raised intracranial pressure caused by a reduction in CSF resorption secondary to adhesions in the subarachnoid space. 13 The second theory concerns CSF dynamics. The lumbosacral region is considered the main CSF reservoir, functioning along with the intracranial vascular pool to maintain constant intracranial pressure. Any changes of this balanced system (e.g., spinal tumors) will lead to increased intracranial pressure and papilledema even without ventriculomegaly in a pseudotumor-like condition. 8

The uncommon association between hydrocephalus and intraspinal tumors should always be kept in mind in the differential diagnosis of hydrocephalus of unknown etiology for three main reasons: the possibility of neurologic deterioration when the patient is shunted prior to tumor removal, the possibility of resolving the hydrocephalus without shunting by simply removing the tumor, and the possible role of hydrocephalus as an early sign of intracranial metastasis in patients previously operated upon with intramedullary gliomas. 3

## Conclusion

Spinal cord tumors associated with hydrocephalus and papilledema are rare but well described. The diagnosis of these conditions may be difficult or confusing as the symptoms referable to the spinal lesion may be minimal or overlooked. Proper history taking, examination, and investigations are mandatory to diagnose this entity.
